# The structural and proteomic analysis of *Spiroplasma eriocheiris* in response to colchicine

**DOI:** 10.1038/s41598-018-26614-y

**Published:** 2018-06-05

**Authors:** Peng Liu, Jie Du, Jia Zhang, Jian Wang, Wei Gu, Wen Wang, Qingguo Meng

**Affiliations:** 1 Jiangsu Key Laboratory for Microbes & Functional Genomics and Jiangsu Key Laboratory for Aquatic Crustacean Diseases, College of Life Sciences, Nanjing Normal University, 1 Wenyuan Road, Nanjing, 210023 China; 2 Department of Biology, College of Pharmacy and Biological Sciences, University of South China, Hengyang, 421001 P.R. China; 3 Hunan Province cooperative innovation Center for Molecular Target New Drug Study, Hengyang, 421001 P.R. China

## Abstract

*Spiroplasma eriocheiris*, a pathogen that causes mass mortality of Chinese mitten crab *Eriocheir sinensis*, is a wall less bacteria and belongs to the *Mollicutes*. This study was designed to investigate the effects of colchicine on *S*. *eriocheiris* growth, cell morphology, and proteins expression. We found that in the presence of colchicine, the spiroplasma cells lost their helicity, and the length of the cells in the experimental group was longer than that of the control. With varying concentrations of the colchicine treatment, the total time to achieve a stationary phase of the spiroplasma was increased, and the cell population was decreased. The virulence ability of *S*. *eriocheiris* to *E*. *sinensis* was effectively reduced in the presence of colchicine. To expound the toxical mechanism of colchicine on *S*. *eriocheiris*, 208 differentially expressed proteins of *S*. *eriocheiris* were reliably quantified by iTRAQ analysis, including 77 up-regulated proteins and 131 down-regulated proteins. Especially, FtsY, putative Spiralin, and NADH oxidase were down-regulated. F_0_F_1_ ATP synthase subunit delta, ParB, DNABs, and NAD(FAD)-dependent dehydrogenase were up-regulated. A qRT-PCR was conducted to detect 7 expressed genes from the iTRAQ results during the incubation. The qRT-PCR results were consistent with the iTRAQ results. All of our results indicate that colchicine have a strong impact on the cell morphology and cellular metabolism of *S*. *eriocheiris*.

## Introduction

*Spiroplasma* is a group of bacteria belonging to a class *Mollicutes*, which includes *Mycoplasma*, *Phytoplasma* and so on, and featured by small genome sizes and lack of peptidoglycan layer^1–3^. They have a helical cell morphology and swim without flagella when in an appropriate viscous medium^2,4^. Locomotion is by propagation of kink pairs along the cell body from one pole to another end^5^. Some *Spiroplasma* cause serious losses in economically important crops and honeybee cultures, and are pathogens of insects and/or plants^6^. *Spiroplasma eriocheiris* is a novel pathogen causing mass mortality of Chinese mitten crab *Eriocheir sinensis*, causing disastrous effects on aquaculture in China in recent years^7^. *S*. *eriocheiris* was the first *Spiroplasma* species isolated from a freshwater crustacean^8^. *S*. *eriocheiris* performs chemotaxis without the conventional two-component system, the system commonly found in bacterial chemotaxis. The cells are polarized by a tip structure, a dumbbell-shaped core in the tip that is connected by a flat ribbon forming the internal structure of *S*. *eriocheiris*. Sixteen proteins were identified as the components of the internal structure by mass spectrometry, including Fibril protein and four types of MreB proteins^9^. The *Spiroplasma* genomes reported so far do not have orthologs of other bacterial motility systems, but have one tubulin homolog-FtsZ and five to seven homologs of the protein MreB^10^. MreB is related to actin, which is responsible for many eukaryotic motility systems^11^.

Colchicine can be extracted naturally from *Gloriosa superba* L. and *Colchicum autumnale* L. plants^12,13^. It is toxic for eukaryotic cells, and is an example of a class of small, tublin-binding molecules^14^. Colchicine is a fat-soluble alkaloid binding to β-tubulin, hindering its polarization with consequent inhibition of neutrophil chemotaxis and reducing expression of adhesion molecules^15^. Moreover, it is known to be a specific mitotic poison, inhibiting normal division of the chromosomes and thus causing mutation^16^. Prokaryotic bacterial cells differ from the eukaryotic cells of higher plants and animals^17^. Well-documented electron microscopic studies of *Bacillus megaterium* have shown that cell morphology was changed by colchicine treatment^17^. *Staphylococcus* showed a temporary variation in cell and colony morphology when grown in the presence of colchicine^18,19^. But, there is no report about the effect of colchicine on cell morphology and metabolism of wall-less bacteria.

In the present study, we evaluated the morphology alterations and proteomics profile of the colchicine-adapted *S*. *eriocheiris* cells by using differential interference contrast (DIC) microscope and electronic microscope (EM), iTRAQ and real time PCR (RT-PCR). We attempt to explain and establish a significant correlation between the structural and metabolic profile of the *S*. *eriocheiris* undergoing challenge by colchicine.

## Results

### Cell morphology

**Figure 1:**
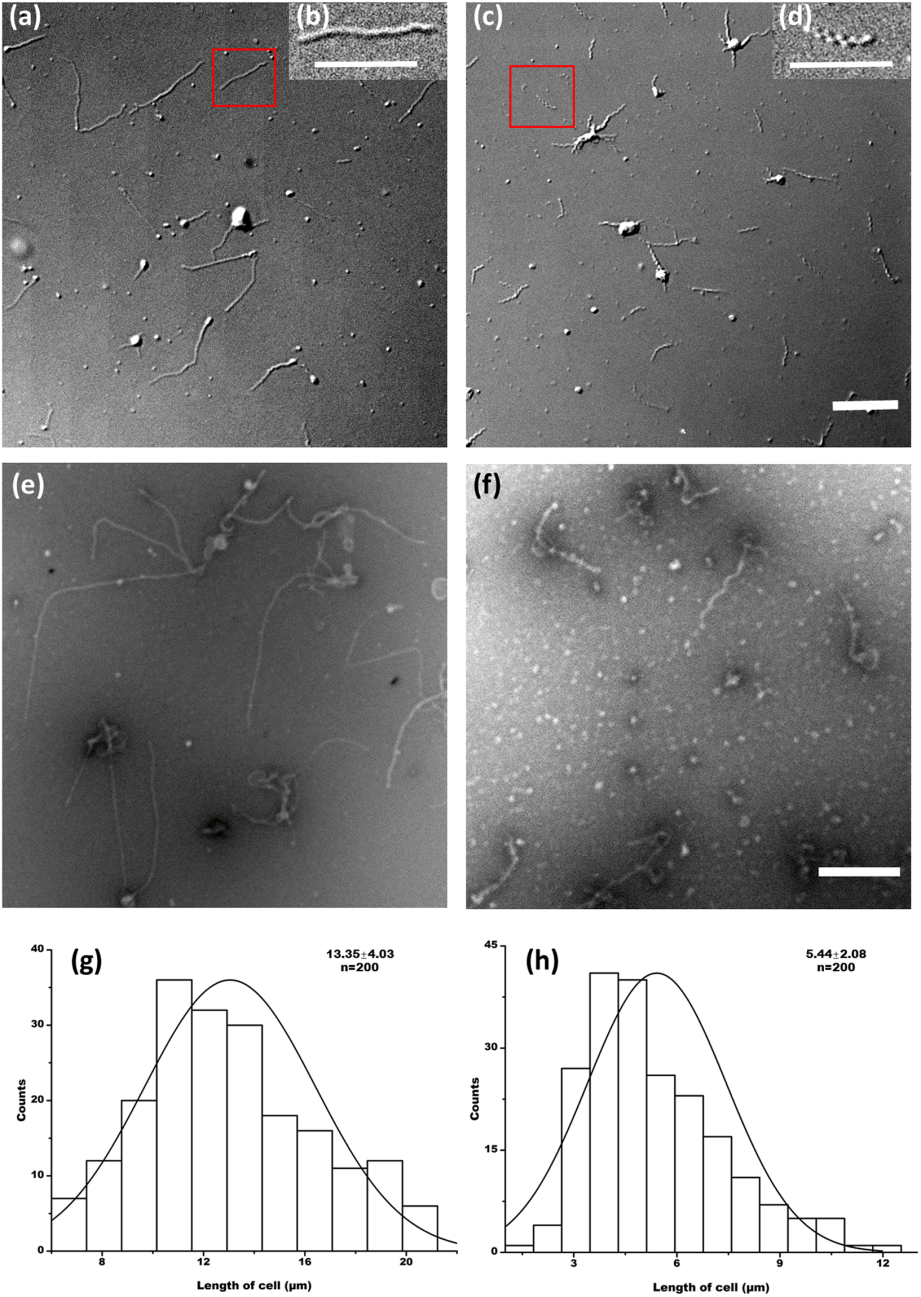
Cell observation by DIC and EM. (**a**) DIC image of *S*. *eriocheiris* cells treated by 3 g/L colchicine. (**b**) A single cell magnified from the red box of figure A. (**c**) DIC image of *S*. *eriocheiris* cells without colchicine treatment. (**d**) A single cell magnified from the red box of figure c (Scale bar,10 μm). Two EM images of *S*. *eriocheiris* cells treated by 3 g/L colchicine, and those from the control, are presented in figures (**e**) and (**f**), respectively (Scale bar: 2 μm). (**g**) The histogram of *S*. *eriocheiris* cell length distribution when treated with 3 g/L colchicine. The average cell length (µm) and measured cell number are shown in the upper right side of the figure. (**h**) Control.

To study the effect of colchicine on *S*. *eriocheiris* cell morphology, 3 g/L colchicine was added into the medium as the final concentrations, and until the cells were in exponential phase. Cells of the *S*. *eriocheiris* collected from the exponential phase of the growth were examined by DIC microscope and EM in the presence of colchicine. In the first instance, we checked the cells by DIC microscope. With 3 g/L colchicine treatment, the *S*. *eriocheiris* cells lost their helical shape, and the length of the cells in the experimental group was longer than that of the control cells (Fig. 1a–d). These results were consistent with EM observations, cells in the colchicine rich medium were longer than those in control conditions, and the treated cells were no longer helically shaped (Fig. 1e,f). *S*. *eriocheiris* cell lengths, when adapted to 3 g/L colchicine, were 13.35 ± 4.03 μm (n = 200) (Fig. 1g). Whereas, under controlled growth conditions, the spiroplasma cell exhibited normal shape, having 5.4 ± 2.08 μm (n = 200) length (Fig. 1h). 3 g/L colchicine-adapted *S*. *eriocheiris* cells were approximately 8 μm longer than control cells.

### Growth kinetics and pathogenic ability

**Figure 2:**
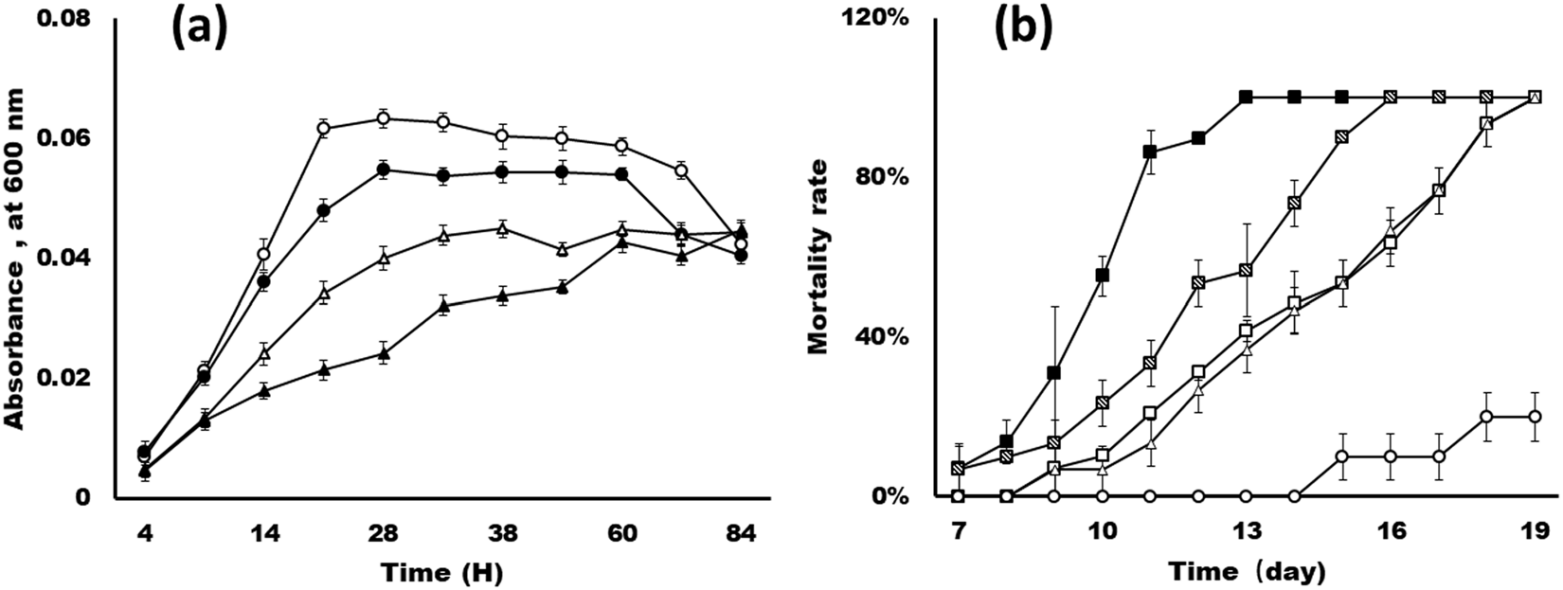
The growth kinetics and pathogenicity of colchicine treated cells. (**a**) Growth of control (open circle) and colchicine (solid circle-1 g/L, open triangle-3 g/L and solid triangle-5 g/L) adapted *S*. *eriocheiris* cells at 30 °C. (**b**) Mortality rate of *E*. *sinensis* after injection with *S*. *eriocheiris* adapted by different concentrations of colchicine. Solid square, hashed square, open square and hatched triangle represent 0, 1, 3, and 5 g/L adapted *S*. *eriocheiris*, respectively. Open circle represents control, *E*. *sinensis* were injected with PBS.

Also, the growth kinetics of *Spiroplasma* were studied with the selective forces of colchicine, when different concentrations of colchicine (0, 1, 3, and 5 g/L) were added into the medium as the final concentrations. The cells were cultured for 24 h, until the cells were in exponential phase. A significant difference was noticed between the growth rates of un-adapted control and colchicine-adapted bacteria (Fig. 2a). Under optimal conditions, the total time taken by the bacteria to achieve a stationary phase was 18 h. The maximum concentration of colchicine acceptable for adaptation of *S*. *eriocheiris* cells was determined to be 5 g/L. A gradual shift was noticed in the generation time for colchicine adapted cells. With reference to control un-adapted cells, 5 g/L colchicine-adapted bacterial cells required 60 h to attain the steady state, but the un-adapted cells required only 18 h. At the same time, the virulence ability of *S*. *eriocheiris* was investigated. After 0, 1, 3, and 5 g/L colchicine-adapted cells were washed by PBS, the cells were injected into *E*. *sinensis*, and the mortality rates of all groups were counted. *E*. *sinensis* died 7_days after the injection of the 0 g/L colchicine-adapted *S*. *eriocheiris*, and reached 100% mortality rate after 13 days. When the *E*. *sinensis* was injected with the 5 g/L colchicine-adapted cells, the *E*. *sinensis* reached 100% mortality rate after 19 days’ post *S*. *eriocheiris* injection. The results show that the mortality rates were decreasing with the increase of colchicine adaptation of the bacterial cells. It means that the virulence ability of colchicine adapted cells was effectively and gradually reduced with the increase of colchicine concentration (Fig. 2b).

### iTRAQ quantification

**Figure 3:**
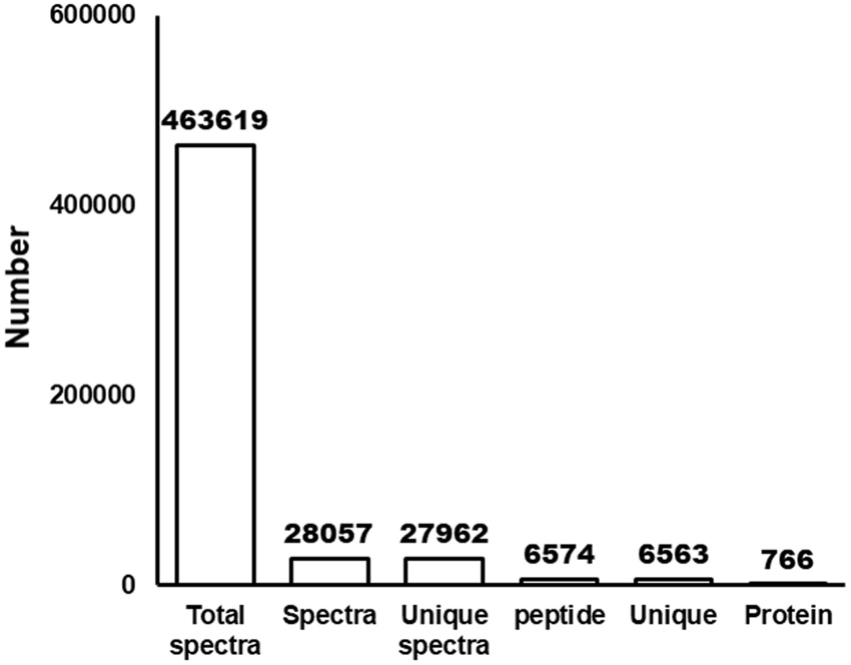
Basic information statistics of proteome resulting from iTRAQ. Total spectra are the secondary mass spectrums, and spectra are the secondary mass spectrums after quality control. Unique peptide is the identified peptides, which belongs only to a group of proteins, and protein is identified by Mascot 2.3.02 software.

**Figure 4:**
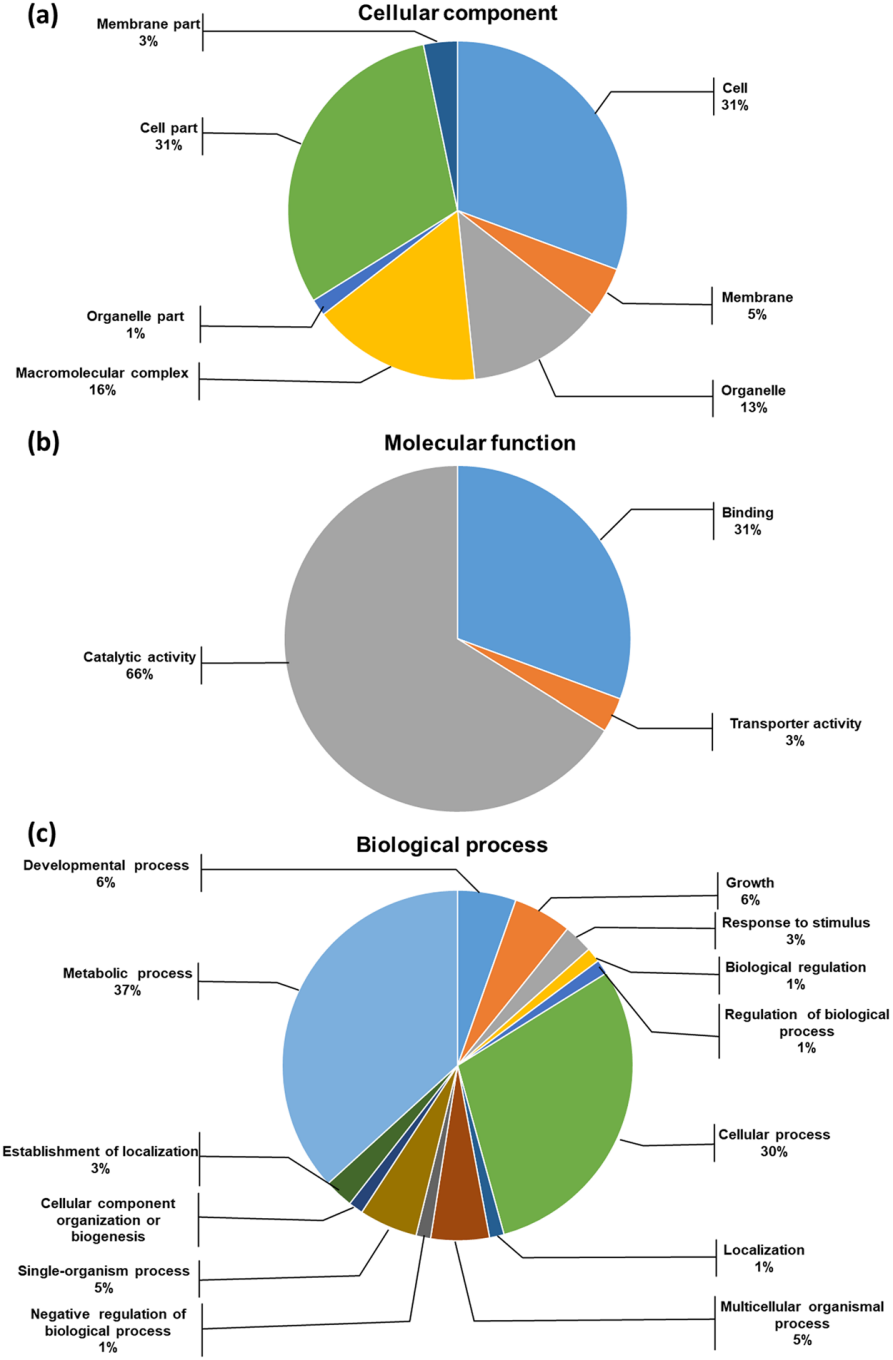
Gene ontology (GO) analysis of total proteins in *S*. *eriocheris*. (**a**) Cellular component, (**b**) molecular function and (**c**) biological process.

All MS/MS spectra were processed by using Mascot software. As shown in Fig. 3, iTRAQ analysis of *S*. *eriocheiris* proteome showed 28057 queries in the database (463,619 sequences), and resulted in 766 identified proteins in Mascot. Gene ontology (GO) analysis of total proteins in *S*. *eriocheiris* was based on cellular component (Fig. 4a), molecular function (Fig. 4b), and biological process (Fig. 4c). We analyzed the differential proteins by iTRAQ quantification. Using a 1.2-fold increase or decrease in protein expression as a benchmark for a physiologically significant fold change, 208 differentially expressed proteins were reliably quantified by iTRAQ analysis, including 77 up-regulated proteins (Table S1) and 131 down-regulated proteins (Table S2) subsequent to colchicine stimulation. In the up-regulated proteins, 5 proteins were involved in energy metabolism processes; 17 proteins were DNA replication and translation related proteins; 6 proteins were related to transport system proteins and transferase; 11 proteins were glycometabolism proteins; 11 proteins were involved in amino acid and protein metabolism processes; 3 proteins were oxidoreductases and 23 proteins were listed as unknown/hypothetical proteins. The up-regulated proteins included: F_0_F_1_ ATP synthase subunit delta (ATP H) (ACCESSION: AHF57252) with 2.386-fold change, Ribonucleotide-diphosphate reductase beta subunit (ACCESSION: AHF57904) with 3.621-fold change, and Chromosome partitioning protein ParB (ACCESSION: AHF57357) with 1.241-fold change. In addition, a Putative NAD(FAD)-dependent dehydrogenase (ACCESSION: AHF57464) was up regulated with a fold change of 1.274, etc.

Of the down-regulated proteins, 12 proteins were grouped within the ribosomal proteins; 7 proteins were involved in energy metabolism processes; 9 proteins were related to carbohydrates, and 13 proteins were involved in amino acid and protein metabolism; 18 proteins were DNA replication and cell division related proteins; 2 were ionic regulation related proteins; 6 proteins were oxidoreductases; 13 proteins were involved in transport systems and served as transferases; 5 proteins were related to lipoprotein and lipid metabolism; and finally, 45 proteins were in the unknown/hypothetical protein category. Especially, the cell division FtsY (ACCESSION: AHF57275) was 0.783-fold down-regulated. FtsY, the prokaryotic signal recognition particle receptor homologue, is essential for biogenesis of membrane proteins^20^. Furthermore, other down-regulated proteins included: lipoprotein-putative Spiralin (ACCESSION: AHF58284) with 0.664-fold change and NADH oxidase (ACCESSION: AHF57728) with 0.622-fold change.

### RT-PCR analysis of the mRNA

In order to provide additional mRNA transcript level information of *S*. *eriocheiris* and validate the iTRAQ result, we performed qRT-PCR on some selected target gene expressions in both the experimental group and control group after 1, 3, 5, 7, 9, and 11 h incubation using 3 g/L colchicine stimulation. In additional, gene expression was investigated when cells were stimulated by using different concentrations (0, 1, 3, and 5 g/L) of colchicine. We measured the mRNA transcription levels of 7 proteins, including 4 genes of up-regulated proteins: F_0_F_1_ ATP synthase subunit delta (ATP H), Chromosome partitioning protein ParB, DNABs (ACCESSION: AHF58167), and Putative NAD (FAD)-dependent dehydrogenase and 3 down–regulated proteins: Cell division related protein FtsY, putative Spiralin, and NADH oxidase.

**Figure 5:**
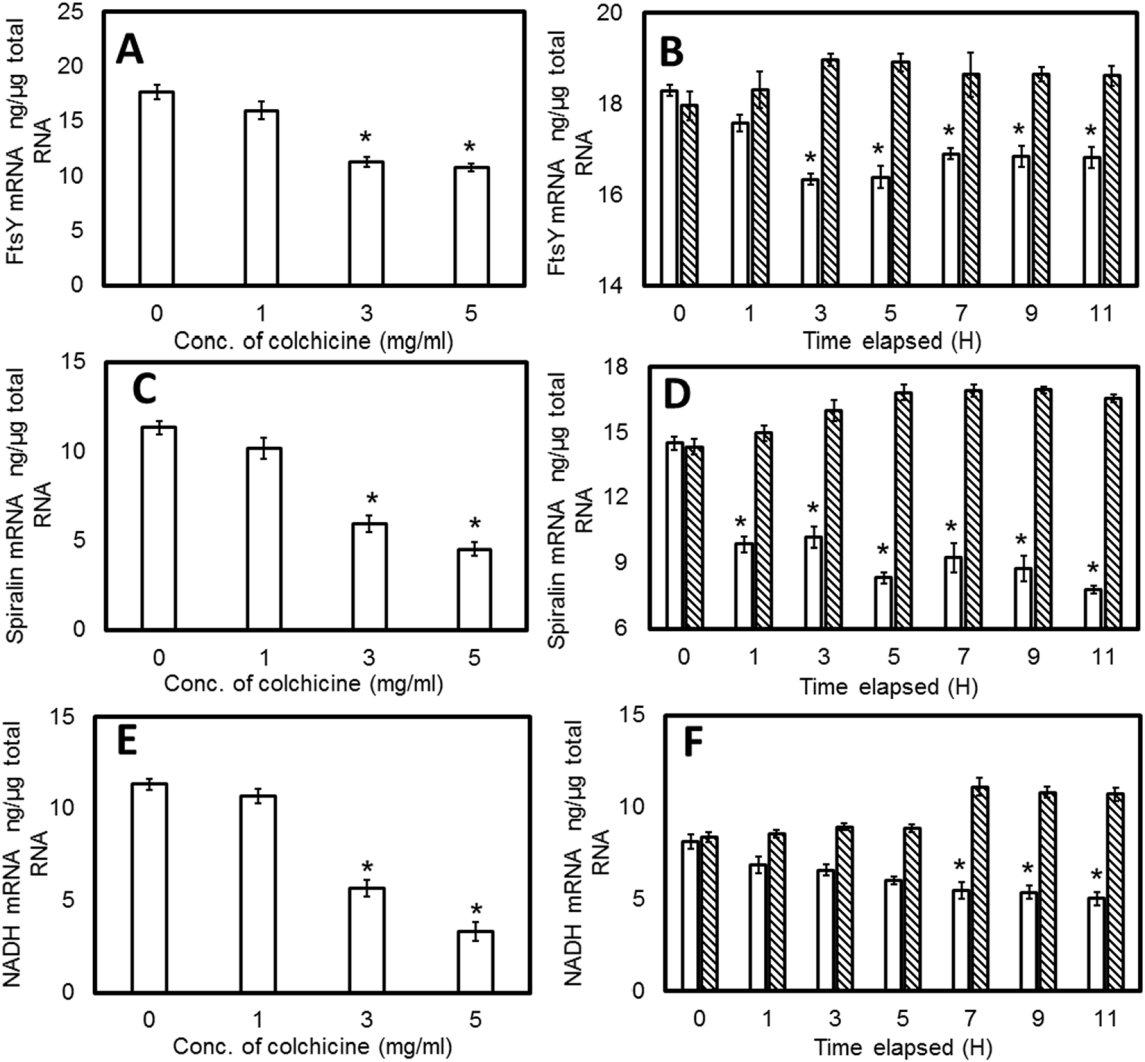
qRT-PCR analysis of down-regulated gene expression of challenged *S*. *eriocheiris*. (**a,c** and **e**) represent FtsY, Spiralin, and NADH oxidase mRNA expression at different concentrations of colchicine challenge; the mRNA expression data under different challenge times (0, 1, 3, 5, 7, 9, and 11 h) are presented in (**b**,**d** and **f**), respectively. Each column represents the mean value with standard error bars based on three samples. Statistical significance (*P* < 0.05) is indicated with an asterisk (*). The open columns indicate gene expressions when challenged with 3 g/L colchicine and hatched as control.

As shown in Fig. 5, we investigated the gene expression of 3 down-regulated genes: FtsY, putative Spiralin and NADH oxidase. When stimulated with colchicine, the gene expression of those three genes decreased concomitant with the increase of colchicine concentration. When the concentration of colchicine was 3 g/L and 5 g/L, the gene expression was significantly lower compared to the 0 g/L treatment (Fig. 5c). During the time course of 3 g/L colchicine stimulation, the three down-regulated genes showed a tendency to decrease over time. The first response gene was putative Spiralin, it showed a significant difference compared to the control group after 1 h of colchicine stimulation (Fig. 5d). But the gene expression of FtsY (Fig. 5b) and NADH oxidase (Fig. 5f) showed a significant difference with the control group after 3 and 7 h of colchicine stimulation, respectively.

**Figure 6:**
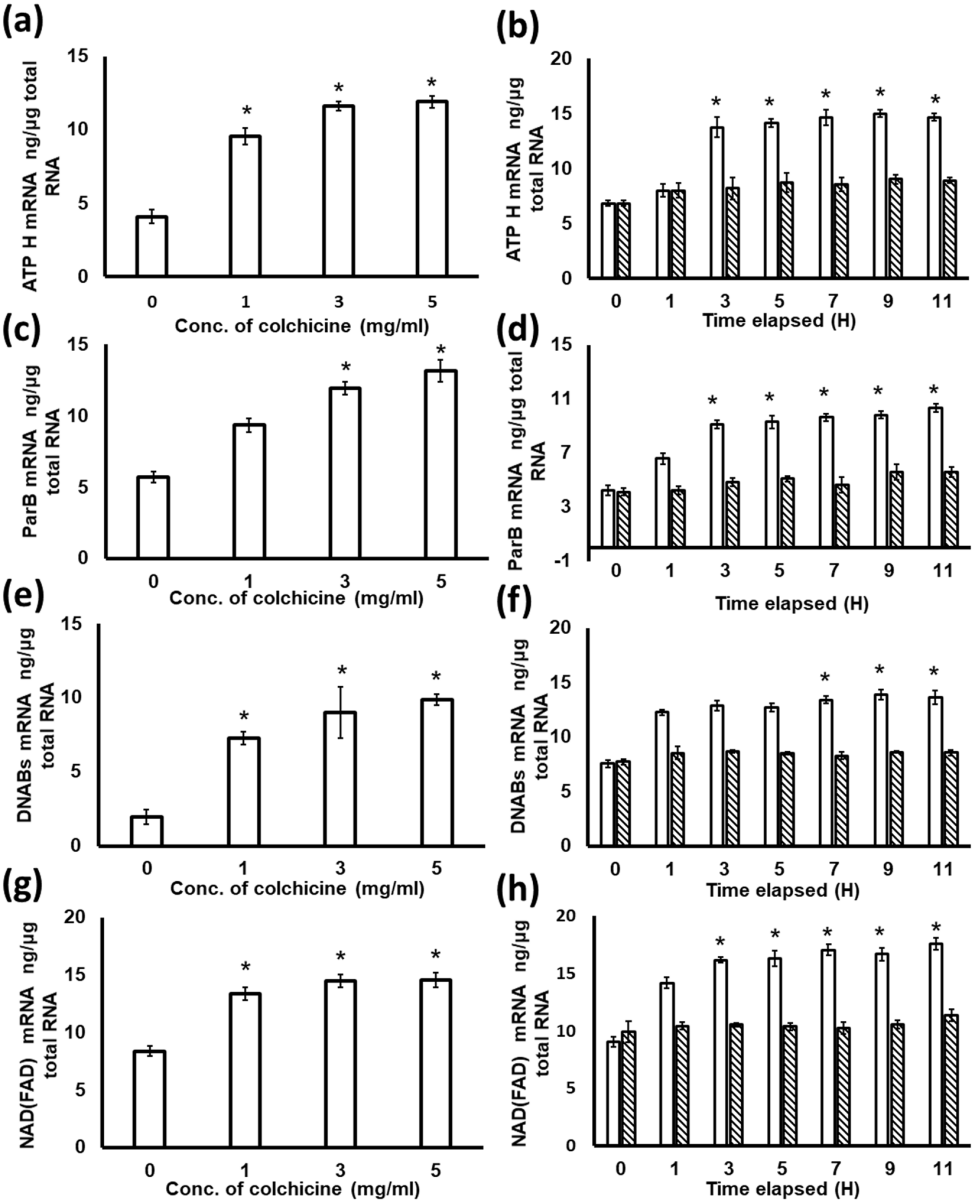
Analysis of up-regulated gene expressions of challenged *S*. *eriocheiris* using qRT-PCR. (**a**,**c**,**e** and **g**) represent ATP H, ParB, DNABs and NAD(FAD) mRNA expression at different concentrations of colchicine challenge; the mRNA expression under different challenge times (0, 1, 3, 5, 7, 9, and11 h) are presented in (**b**,**d**,**f** and **h**), respectively. Each column represents the mean value from three samples with the standard error bars. Statistical significance (p < 0.05) is indicated with an asterisk (*). The open columns indicate gene expressions when challenged with 3 g/L colchicine and hatched as control.

As shown in Fig. 6, we investigated the gene expressions of 4 up-regulated genes (ATP H, ParB, DNABs and NAD (FAD) dependent dehydrogenase) under the stimulation of colchicine. The genes ATP H (Fig. 6a), DNABs (Fig. 6e) and NAD (FAD) dependent dehydrogenase (Fig. 4g) showed a rapid increased response when the concentration of colchicine was 1 g/L, and were significantly different from the response with 0 g/L. But, ParB showed a significant difference from the control preparation when the concentration of colchicine was higher than 3 g/L (Fig. 6c). Under the stimulation of 3 g/L colchicine, the 4 up-regulated genes showed an increased response over time after 3 h of colchicine stimulation. ATP H (Fig. 6b), ParB (Fig. 6d) and NAD (FAD) dependent dehydrogenase (Fig. 6h) had a significant increase up to 7 h after the colchicine stimulation when compared with time 0 h. DNABs had a significant increase after 7 h of the colchicine stimulation (Fig. 6f). To sum up, all the results by RT-PCR analyses were consistent with the iTRAQ analyses.

## Discussion

### The cell morphology alternated by colchicine

The aim of this work was to evaluate the effect of colchicine on the *S*. *eriocheiris* cell morphological alterations and changes in the proteomic profile. Colchicine is an example of a class of small molecules that bind to tubulin and inhibits its polymerization and thus is toxic to bacteria^14,16,17^. However, scanty reports are available on structural and metabolic alterations of *B*. *megaterium* under colchicine treatment^21,22^. Kashyap Kumar Dubeya demonstrated that the cellular architectural structure of *B*. *megaterium* was changed when cells were challenged by colchicine. Colchicine adapted cells lost their helical cell morphology and became longer than control cells^17^. It is well established that the plasma membrane is composed of a phospholipid bilayer, including different types of proteins, and the length of phospholipid chain is almost constant, so the thickness of the plasma membrane may vary depending on the amount of proteins. The reduction in thickness of the bacterial cell membrane is possible due to loosening of weakly immobilized membrane protein components, or inhibition of membrane protein synthesis^23^. According to the iTRAQ results, 3% membrane part and 5% membrane proteins were alliterated (Fig. 4a). Therefore, alterations in cell morphology may be caused by reduction of the amount of membrane proteins, particularly in bacterial cells adapted to high concentrations of colchicine^24^. Even if, no cytoskeleton protein was found from the iTRAQ results, the cytoskeleton of *S*. *eriocheiris* may lose the bind point on the membrane. 31% binding proteins in *S*. *eriocheiris* cells were changed by colchicine challenge (Fig. 4b).

### Cell division and energy metabolism process of the *S*. *eriocheiris* may be disturbed by colchicine

iTRAQ profiling, a good method for protein measurement, has been shown to be very useful for quantifying changes in plasma proteins in bacteria^25^. For example, Redding *et al*. used iTRAQ proteomics to study the proteomic profile of *Desulfovibrio vulgaris* under nitrate stress^26^. Evans *et al*. investigated the regulation and biosynthesis of bioactive compounds using iTRAQ and proposed a correlation between phosphate and iron regulation and the biosynthesis of bioactives in *Pseudoalteromonas tunicata*^27^. In this study, the alteration proteins were reliably quantified by using iTRAQ analysis. Theose proteins are involved in the process of energy metabolism; glycometabolism; protein and amino acid metabolism; DNA transcription and translation or cell division, and other processes.

The cell morphology and motility of *Spiroplasmas* are mediated by the internal cytoskeleton^11,28^. It has been reported that the cytoskeleton of *S*. *eriocheiris* contains 16 proteins, including Fibril and four types of Mrebs^9^. But no cytoskeletal proteins were detected by the iTRAQ analysis. The changes of metabolism of *S*. *eriocheiris* might have resulted in cell length increase and losing cell helicity as evident from iTRAQ analysis. The colchicine adapted cells showed longer cell length than controls. This may be caused by the changes of DNA transcription and translation or by changes in cell division proteins. Based on iTRAQ results, there were changes in protein responses, including: cell division related proteins FtsY (0.738-fold down-regulated), ParB (1.24-fold up-regulated), and DnaBs (2.08 -fold up-regulated). FtsY, the prokaryotic signal recognition particle receptor homologue, is essential for biogenesis of membrane proteins and cell division. Also, FtsY is indeed essential for expression of integral membrane proteins in *E*. *coli*^20,29^. In unicellular bacteria, ParB is a broadly conserved molecular mechanism for plasmid partitioning and chromosome segregation^30^, the ParA and ParB proteins segregate chromosomes and coordinate this process with cell division and chromosome replication^30^. DnaB, a chromosome replication initiation/membrane attachment protein, is an enzyme in bacteria which opens the replication fork during DNA replication^31^. Totally, 17 up-regulated proteins and 18 down–regulated proteins were related to bacterial DNA transcription and translation or cell division. So *Spiroplasma* cells could not proceed through normal cell division when challenged with colchicine, thus resulting in increased cell length.

### Pathogenicity and growth speed of the *S*. *eriocheiris* was inhibited by colchicine

Under the stimulation of different concentrations of colchicine, pathogenicity and growth kinetics were inversely correlated. Probably, these responses were caused by alterations of some energy metabolism proteins and pathogenic factor proteins. According to iTRAQ results, 13 transferase and transport proteins were down-regulated. 7 down-regulated proteins and 4 up-regulated proteins were related to energy metabolism. These energy-related proteins include ATPases, a class of enzymes that catalyze the decomposition of ATP into ADP and a free phosphate ion. Transmembrane ATPases import many of the metabolites necessary for cell metabolism and export toxins, wastes, and solutes^32,33^. Therefore, energy transformation in colchicine adapted cell was altered and the growth rate was decreased. At the same time, as the concentration of colchicine was increased, the pathogenic ability decreased. This may be caused by reduction of a pathogenic factor. For example, Spiralin is one of the most thoroughly characterized *S*. *citri* membrane lipoprotein proteins^34^. It is important for *S*. *citri* GII3 to adhere to and invade insect cells^35^. It might be predicted that the process of responding to colchicine severely affects the metabolic profile and morphology of the *S*. *eriocheiris*. There is a correlation between the structural and metabolic profile of the cell. This phenomenon is consistent with the changes of *B*. *megaterium* cell under colchicine challenge^17^.

## Conclusion

Our results indicate that colchicine may have an effect on the cell morphology and cellular metabolism of *S. eriocheiris*. There are 208 differentially expressed proteins in the *S*. *eriocheiris* proteomics at 24 h post colchicine incubation. Most of the differential proteins are related to changes in intracellular metabolic processes. Based on the evidence of differentially expressed proteins during colchicine treatments, we can speculate about the structural and metabolic changes of *S*. *eriocheiris*. In the presence of colchicine, the* S. eriocheiris* lost its helicity, resulting in a longer cell length than that of the control group. The energy metabolism, cell division process and pathogenicity were interrupted by colchicine. Overall, the novelty of the study is that this is the very first time that the effect of colchicine has been reported on the wall less bacterial cell. Furthermore, our study has shown a correlation between the colchicine-induced structural alterations with the proteomic profile of the bacterium.

## Methods

### Bacterial Strains and Culture conditions

The strain, *S*. *eriocheiris* was isolated from haemolymph of the Chinese mitten crab, *E*. *sinensis*^36^. The optimum growth occurs at 30 °C in R2 broth. Pathogenicity ability were estimated by counting the mortality after the injection of *S*. *eriocheiris* cells into *E*. *sinensis*. At the initial stages of adaptation, 0.1 g/L colchicine was used as a selective force. Under such conditions the *S*. *eriocheiris* cells were grown for about five generations till the specific growth was obtained similar to the control non-adapted bacterial cells. At the end of each generation the bacterial cultures were evaluated. The ability of the bacterial culture to grow in contact with different selective concentrations (0, 1, 3, and 5 g/L) of colchicine was examined when it was added into R2 medium.

### Cell observation and measurement

3 g/L colchicine was added into the medium as the final concentrations, and then the cells were cultured for 24 h, until the cells were in exponential phase. Cultured cells were centrifuged at 11,000 × g, for 30 min at 4 °C and the pellet was resuspended with PBS buffer (75 mM sodium phosphate (pH 7.3) and 68 mM NaCl) with 0.6% methyl cellulose. The cell was video recorded with a DIC microscope. The videos were analyzed by Image J ver.1.37 v (http://rsb.info.nih.gov/ij/). 200 cell lengths were measured from videos. As for the EM observation, the cultured cells suspended in PBS equivalent to the original density were placed onto an EM grid, and incubated for 10 min at room temperature (RT). The excess fluid on the EM grid was removed and the preparation was stained with ammonium molybdate for 1 min.

### Experimental bacterial infection and the growth kinetics evaluation

Experimental *E*. *sinensis* (50 ± 3 g) were purchased from a market in Nanjing, China, and cultivated in 10 L tanks. Only healthy *E*. *sinensis* were selected and randomly divided into five groups. *S*. *eriocheiris* cells were washed with PBS two times and adjusted to a final concentration of 10^8^ /ml *Spiroplasma* cells. The *E*. *sinensis* in each group (30 individuals) received an injection of 100 μL washed *S*. *eriocheiris*, that were each adapted by treatment with 0, 1, 3, and 5 g/L colchicine, respectively. Thirty *E*. *sinensis*, receiving an injection of 100 μL PBS individually, were used as a control group. After treatment with different concentrations of colchicine adapted *S*. *eriocheiris*, or with PBS as a control, the mortality rates of all groups were determined. As for the growth kinetics evaluation, *Spiroplasma* cells were cultured in R2 medium containing different concentrations of colchicine without phenol red stain. The relative cell number was measured by a spectrophotometer at a wavelength of 600 nm.

### Protein preparation

*S*. *eriocheiris* cell samples treated with 0 or 3 g/L colchicine solutions were washed by PBS and centrifuged at 11,000 × g, for 30 min at 4 °C three times. Three different replicates of *S*. *eriocheiris* cell samples were mixed as one sample, mixed cell samples were ground into powder in liquid nitrogen, extracted with Lysis buffer A (7 M Urea, 2 M Thiourea, 4% CHAPS, 40 mM Tris-HCl, pH 8.5) containing 1 mM PMSF and 2 mM EDTA. After 5 min, 10 mM DTT was added. After sonication and centrifugation, the suspension was mixed well with a 5-fold volume of chilled acetone containing 10% TCA and incubated at −20 °C overnight. After centrifugation at 4 °C, 30,000 × g, the precipitate was washed with chilled acetone three times. The pellet was air-dried and dissolved in Lysis Buffer B (7 M Urea, 2 M Thiourea, 4% NP40, and 20 mM Tris-HCl, pH 8.5). The suspension was sonicated at 200 W for 15 min and centrifuged at 4 °C, 25,000 × g for 20 min. To reduce disulfide bonds in proteins of the supernatant, 10 mM DTT was added and incubated at 56 °C for 1 h. Subsequently, 55 mM IAM was added to block the cysteines, and incubated for 1 h in the darkroom. The supernatant was mixed well with a 5-fold volume of chilled acetone for 2 h at −20 °C. After centrifugation, the pellet was air-dried for 5 min, dissolved in 200 μL of 0.5 M TEAB (Applied Bio-systems, Italy) and sonicated at 200 W for 15 min. Finally, samples were centrifuged at 4 °C, 25,000 × g for 20 min. The supernatant was transferred to a new tube and quantified using a 2-D Quant Kit (GE Healthcare). The proteins in the supernatant were kept a −80 °C for further analysis.

### iTRAQ labeling and SCX fractionation

The iTRAQ assays were performed as described previously with minor modification^37^.Total protein (100 μg), taken from each sample solution, was digested with Trypsin Gold (Promega, USA) with the ratio of protein: trypsin = 20: 1 at 37 °C for 4 h. After trypsin digestion, peptides were dried by vacuum centrifugation. Peptides were reconstituted in 0.5 M TEAB and processed according to the manufacturer’s protocol for 8-plex iTRAQ reagent (Applied Biosystems). The proteins from the non-treated and treated samples were labeled with 115 and 114, respectively. The labeled peptide mixtures were then pooled and dried by vacuum centrifugation. The labeled samples were pooled and purified using a strong cation exchange chromatography (SCX) column (Phenomenex, USA), and separated by liquid chromatography (LC) using a LC-20AB HPLC Pump system (Shimadzu, Japan). The iTRAQ-labeled peptide mixtures were reconstituted with 4 mL buffer A (25 mM NaH_2_PO_4_ in 25% ACN, pH 2.7) and loaded onto a 4.6 × 250 mm Ultremex SCX column containing 5 mm particles (Phenomenex). The peptides were eluted at a flow rate of 1 mL/min with a gradient of buffer A for 10 min, 5–60% buffer B (25 mM NaH2PO4, 1 M KCl in 25% ACN, pH 2.7) for 27 min, and 60–100% buffer B for 1 min. The system was then maintained at 100% buffer B for 1 min before equilibrating with buffer A for 10 min prior to the next injection. Elution was monitored by measuring the absorbance at 214 nm, and fractions were collected every 1 min. The eluted peptides were pooled into 20 fractions, desalted with a Strata X C18 column (Phenomenex) and vacuum-dried.

### LC-ESI-MS/MS analysis based on Triple TOF 5600

Each fraction was resuspended in buffer C (5% ACN, 0.1% FA) and centrifuged at 20,000 × g for 10 min, the final concentration of peptide was about 0.5 g/L on average. 10 mL of supernatant was loaded on a LC-20AD nanoHPLC (Shimadzu, Japan) by the autosampler onto a 2 cm C18 trap column. Then, the peptides were eluted onto a 10-cm analytical C18 column packed in-house. The samples were loaded at 8 mL/min for 4 min, then a 35 min gradient was run at 300 nL/min starting from 2 to 35% buffer D (95% ACN, 0.1% FA), followed by 5 min linear gradient to 60%, then followed by a 2 min linear gradient to 80%, and maintenance at 80% buffer D for 4 min, and finally returned to 5% for 1 min.

Data acquisition was performed with a Triple TOF 5600 System fitted with a Nanospray III source (AB SCIEX) and a pulled quartz tip as the emitter (New Objectives, MA). Data was acquired using an ion spray voltage of 2.5 kV, curtain gas of 30 psi, nebulizer gas of 15 psi, and an interface heater temperature of 150. The MS was operated with a RP of greater than, or equal to, 30,000 FWHM for TOF MS scans. For IDA, survey scans were acquired in 250 ms and as many as 30 product ion scans were collected if they exceeded a threshold of 120 counts per second (counts/s). Total cycle time was fixed at 3.3 s. The Q2 transmission window was 100 Da for 100%. Four time bins were summed for each scan at a pulser frequency value of 11 kHz through monitoring of the 40 GHz multichannel TDC detector with a four-anode channel detect ion. A sweeping collision energy setting of 35 ± 5 eV, coupled with iTRAQ adjust rolling collision energy, was applied to all precursor ions for collision-induced dissociation. Dynamic exclusion was set for 1/2 of peak width (15 s), and then the precursor was refreshed to the exclusion list.

### Data analysis

Raw data files acquired from the Orbitrap were converted into MGF files using Proteome Discoverer 1.2 (PD 1.2, Thermo) and the MGF files were searched. Protein identification was performed by using Mascot search engine (Matrix Science, UK). The parameters used included: Gln-> pyro-Glu (N-term Q), Oxidation (M), Deamidated (NQ) as the potential variable modifications, and Carbamidomethyl (C), iTRAQ8plex (N-term), and iTRAQ8plex (K) as fixed modifications. The charge states of peptides were set to +2 and +3. Then protein identification was performed using the genome sequence of *S*. *eriocheiris* (1242 sequences). To reduce the probability of false peptide identification, only peptides with significance scores (≧20) at the 99% confidence interval by a Mascot probability analysis greater than “identity” were counted as identified. And each confident protein identification involved at least one unique peptide. For protein quantization, a protein must have contained at least two unique peptides. The quantitative protein ratios were weighted and normalized by the median ratio in Mascot. We only used ratios with *P*-values < 0.05, and only changes of >1.2 fold were considered as significant. Functional annotations of the proteins were conducted using Blast2GO program against the non-redundant protein database (NR; NCBI).

### Real-time PCR

The absolute real-time standard curve of focused genes was prepared according to the described method^38^. After treatment with different concentrations of colchicine, or for different lengths of time using 3 g/L colchicine stimulation, *S*. *eriocheiris* cells were collected by centrifugation 11,000 × g, 30 min, 4 °C. The total RNAs from *S*. *eriocheiris* cells were extracted from the samples of the different groups. After reverse-transcription into cDNA, real time PCR was carried out to measure the expression levels of focused genes. The primers of amplified genes were listed in Table 1. Data were analyzed using the SPSS general linear models (GLM) procedure (SPSS 22.0, Chicago, IL, USA) to test for significant differences among treatments. If a significant (*P* < 0.05) difference was found, a Duncan’s multiple range test^39^ was used to rank the means. All data are presented as mean ± S.D (standard deviation) of three biological replicates.

**Table 1:** The primers used for real-time PCR in the experiment.

Name	Sequence (5′–3′)
*S*. *eriocheiris* FtsY-F	CCAAAGTCGCGCCAAAACA
*S*. *eriocheiris* FtsY-R	CGGAAGGTATCACCCGCAA
*S*. *eriocheiris* putative Spiralin-F	ACCGTAACAGTACAGGCTCA
*S*. *eriocheiris* putative Spiralin-R	ACCACCACTTTGAATTGCCG
*S*. *eriocheiris* NADH oxidase -F	TTATTGCGACTGGTGCTCGT
*S*. *eriocheiris* NADH oxidase -R	CCAGCGCCAACAATTACGAC
*S*. *eriocheiris* ATP H -F	AGAACCATTAACGCAAGAGCA
*S*. *eriocheiris* ATP H -R	TTGACCAGCTAATGTTCCATCAA
*S*. *eriocheiris* dnaBs -F	AATTATTGGGCACCAGGCTCA
*S*. *eriocheiris* dnaBs -R	GGGTTCCCTTTTCCCGCTTTA
*S*. *eriocheiris* ParB -F	TTTAGTCGCGGGAGAACGTC
*S*. *eriocheiris* ParB -R	GGCGTTGGCTTCTTCAATGG
*S*. *eriocheiris* NAD(FAD)-dependent dehydrogenase -F	GTTGATGCTTTCCACGCCAA
*S*. *eriocheiris* NAD(FAD)-dependent dehydrogenase -R	TTGCTAAATGCACTCCGGCT

## Electronic supplementary material

Supplemental Material
