# Supplementary material for: The structural and proteomic analysis of Spiroplasma eriocheiris in response to colchicine
Source: Sci Rep. 2018 Jun 5;8:8577. doi: 10.1038/s41598-018-26614-y (PMC5988712; doi:10.1038/s41598-018-26614-y)
Supplement: Supplementary file 1 — Supplemental Material [file 41598_2018_26614_MOESM1_ESM.pdf]

The structural and proteomic analysis of *Spiroplasma eriocheiris* in response to colchicine

Peng Liu[1,2,3], Jie Du[1], Jia Zhang[1], Jian Wang[1], Wei Gu[1], Wen Wang[1], Qingguo Meng[1]*

[1] Jiangsu Key Laboratory for Microbes & Functional Genomics and Jiangsu Key Laboratory for Aquatic Crustacean Diseases, College of Life Sciences, Nanjing Normal University, 1 Wenyuan Road, Nanjing 210023, China.

[2] Department of Biology, College of Pharmacy and Biological Sciences, University of South China, Hengyang 421001, PR. China.

[3] Hunan Province cooperative innovation Center for Molecular Target New Drug Study. Hengyang 421001, PR. China.

Running title: *Spiroplasma eriocheiris* in response to colchicine

*Corresponding authors: Qingguo Meng, College of Life Sciences, Nanjing Normal University, 1 Wenyuan Road, Nanjing 210023, PR. China. Tel: +86-25-85891955;

E-mail: mlzzcld@aliyun.com.

Table. S1. Up-regulated proteins in *S. eriocheiris* with a 1.2-fold change cultivated with colchicine.

Protein name	Score	Coverage	Peptide	Fold change	Accession
**Energy metabolism proteins**					
FoF1 ATP synthase subunit delta	1123	51.9	8	2.386	AHF57252
FoF1 ATP synthase subunit epsilon	769	45.1	3	1.797	AHF57256
FoF1 ATP synthase subunit alpha	3260	38.2	17	1.717	AHF57253
GMP synthase	1175	39.6	15	1.619	AHF58150
F0F1 ATP synthase subunit A	148	10.3	2	1.592	AHF57249
**DNA transcription and translation proteins**					
Ribonucleotide-diphosphate reductase beta subunit	1176	39.1	12	3.621	AHF57904
Anaerobic ribonucleoside triphosphate reductase	695	18.6	12	2.712	AHF57296
Putative transcriptional regulator	531	44.1	5	2.219	AHF57284
DNABs	93	7.9	3	2.08	AHF58167
C-terminal truncated transcriptional regulator	314	13.7	2	1.921	AHF57609
Hypoxanthine-guanine phosphoribosyltransferase	710	60.9	10	1.858	AHF58151
Putative cytidine deaminase	172	14.5	2	1.831	AHF57927
Putative single-strand DNA-binding protein	464	22.9	3	1.693	AHF57180
Putative DnaD-like replication protein	520	43	8	1.622	AHF58078
DNA-directed RNA polymerase subunit delta	287	23.5	3	1.586	AHF57366
Putative adenine phosphoribosyltransferase	814	57.3	5	1.578	AHF57630
Molecular chaperone DnaJ	2008	41.1	15	1.483	AHF57313
Putative endonuclease	94	20.6	4	1.486	AHF57353
Heat-inducible transcription repressor HrcA	1397	43.7	14	1.35	AHF57310
Chromosome partitioning protein ParB	2583	63.9	16	1.241	AHF57357
Putative DNA primase	967	27.6	13	1.21	AHF57979
**Transferase and transport proteins**					
ABC-type multidrug transport system permease and ATP-binding protein	135	5.4	3	2.003	AHF57828
Putative ABC-type multidrug transport system ATP-binding protein	189	29.8	5	1.357	AHF57882
ABC-type iron-sulfur cluster assembly transport system permease protein	1411	50.6	20	1.316	AHF58272
PTS system phosphocarrier protein HPr	1831	48.3	3	1.307	AHF57452

Putative PTS system IIB component	1883	70.4	5	1.237	AHF58205
ABC-type cobalt transport system ATP-binding protein	1754	51.2	13	1.226	AHF57401
**Glycometabolism proteins**					
Acetolactate decarboxylase	551	22	4	1.865	AHF58324
Citrate lyase beta subunit	409	21.8	5	1.842	AHF57771
Pyruvate dehydrogenase E3 (dihydrolipoamide dehydrogenase) component	4077	56.6	18	1.442	AHF57469
Putative phosphate acetyltransferase	3014	43.3	12	1.434	AHF58102
Citrate lyase alpha subunit	375	12.9	6	1.379	AHF57770
Pyruvate dehydrogenase E1 component beta subunit	3149	66	17	1.389	AHF57467
Pyruvate dehydrogenase E1 component alpha subunit	3779	76.8	24	1.386	AHF57466
acetolactate synthase	2681	42.2	21	1.366	AHF58325
Putative 2,3-bisphosphoglycerate-independent phosphoglycerate mutase	3282	55	18	1.33	AHF58044
Fructose-bisphosphate aldolase	5931	74	13	1.29	AHF58326
Mannose-6-phosphate isomerase	657	30.8	8	1.206	AHF57226
**Protein and amino acid metabolism proteins**					
Putative amino acid permease	69	5.4	2	2.289	AHF57614
Putative zinc metallopeptidase	94	12.7	3	2.021	AHF57889
Tyrosyl-tRNA synthetase	1547	47.8	14	1.556	AHF58207
Putative methionine aminopeptidase	109	6.1	1	1.369	AHF57440
Putative HAD superfamily hydrolase	408	39.1	9	1.355	AHF57323
Putative tRNA/rRNA Methyltransferase	250	26.4	5	1.346	AHF57348
ATP-dependent serine protease La	6495	51.5	36	1.34	AHF57593
Putative lipoate-protein ligase A	583	26.1	7	1.297	AHF57465
tRNA pseudouridine synthase A	110	13.5	3	1.246	AHF57403
Putative tRNA modification GTPase	560	24.1	9	1.235	AHF58345
16S rRNA processing protein	93	12.8	2	1.232	AHF58160
**Oxidoreduction proteins**					
Putative alkyl hydroperoxide reductase	426	49.3	6	1.456	AHF57576
Putative NAD(FAD)-dependent dehydrogenase	3551	55.3	16	1.274	AHF57464
Putative 5-formyltetrahydrofolate cyclo-ligase	289	39.8	6	1.214	AHF58271
**Hypothetical proteins**					
Hypothetical protein	247	24.4	3	2.317	AHF57811
Hypothetical protein	219	15.5	2	2.17	AHF57563
Hypothetical protein	502	26.6	12	1.967	AHF57851
Conserved hypothetical protein	57	18.5	2	1.92	AHF57951

Conserved hypothetical protein	319	47.7	3	1.844	AHF57393
Conserved hypothetical protein	73	29.7	3	1.833	AHF58174
Putative lipoprotein	154	17.5	5	1.662	AHF57524
Hypothetical protein	198	16.8	2	1.416	AHF57558
Conserved hypothetical protein	751	22.1	14	1.415	AHF57388
Conserved hypothetical protein	255	23.5	5	1.404	AHF57976
Conserved hypothetical protein	139	20.2	3	1.403	AHF58083
Conserved hypothetical protein	1188	61.2	11	1.366	AHF57699
Conserved hypothetical protein	604	42	6	1.358	AHF57460
Putative transmembrane protein	675	15.5	7	1.351	AHF57303
Conserved hypothetical protein	314	19.6	4	1.344	AHF57230
Putative transmembrane protein	198	12.1	3	1.344	AHF57950
Conserved hypothetical protein	161	16.1	6	1.322	AHF57291
Putative transmembrane protein	48	8.8	1	1.295	AHF57620
Conserved hypothetical protein	270	15.7	4	1.287	AHF57671
Hypothetical protein	838	28.3	8	1.263	AHF57389
Putative transmembrane protein	133	7.9	3	1.232	AHF57793
Putative lipoprotein	1236	45.7	8	1.245	AHF57832
Conserved hypothetical protein	206	17.5	5	1.219	AHF58211
Conserved hypothetical protein	682	23	6	1.207	AHF57512

Table. S2. Down-regulated proteins in *S. eriocheiris* with a 1.2-fold change cultivated with colchicine.

Protein name	Score	Coverage	Peptide	Fold change	Accession
**Ribosome proteins**					
50S ribosomal protein L24	313	26.4	3	0.526	AHF57425
Ribosome-binding factor A	1066	62.8	7	0.552	AHF57924
50S ribosomal protein L28	157	23.1	2	0.582	AHF58121
30S ribosomal protein S17	397	45.9	4	0.622	AHF57423
30S ribosomal protein S13	318	28.9	5	0.681	AHF57436
50S ribosomal protein L29	942	30.1	9	0.774	AHF57422
30S ribosomal protein S8	1486	65.9	9	0.798	AHF57428
50S ribosomal protein L10	1642	62.6	9	0.809	AHF58311
50S ribosomal protein L18	734	47.5	6	0.813	AHF57430
30S ribosomal protein S10	1442	43.6	4	0.82	AHF57413
30S ribosomal protein S9	921	33.1	4	0.827	AHF58290
50S ribosomal protein L20	1024	41.2	5	0.832	AHF57837
**Energy metabolism proteins**					
Putative ATP/GTP-binding protein	524	19	12	0.685	AHF58047
Cation transport ATPase	1249	18.9	15	0.67	AHF58203
Putative pantothenate kinase	279	29.8	6	0.724	AHF57913
GTP-binding protein EngA	1158	37	13	0.751	AHF57491

Bifunctional riboflavin kinase/FAD synthetase	578	22.8	6	0.778	AHF57911
Putative ribosome-associated GTPase	172	18.8	5	0.802	AHF58124
Magnesium transport ATPase	341	5.8	4	0.821	AHF57514
**Glycometabolism proteins**					
Galactose-6-phosphate isomerase subunit LacB	393	37.3	5	0.317	AHF57192
Galactose-6-phosphate isomerase subunit LacA	361	43.4	6	0.407	AHF57191
Fructose/tagatose bisphosphate aldolase	332	26.3	5	0.532	AHF57184
Glucose-6-phosphate isomerase	1301	37.9	12	0.578	AHF57394
L-lactate dehydrogenase	1381	44.2	12	0.614	AHF57943
Putative transketolase	3259	39	21	0.683	AHF57705
Triosephosphate isomerase	3250	62	11	0.698	AHF58173
Putative dihydrolipoamide acetyltransferase	1885	43	19	0.736	AHF58143
Ribose 5-phosphate isomerase	755	44.8	4	0.782	AHF58195
**Protein and amino acid metabolism proteins**					
Putative Xaa-Pro dipeptidase	913	27.9	9	0.548	AHF57673
Putative Xaa-His dipeptidase	3347	59.7	21	0.639	AHF58016
Putative nitroreductase	935	41.4	6	0.712	AHF57280
Asparaginyl-tRNA synthetase	1180	41.8	17	0.752	AHF57457
S-adenosylmethionine synthetase	233	14.3	6	0.764	AHF57281
Bifunctional phosphopantothenoylcysteine decarboxylase/phosphopantothenate synthase	1098	36.4	13	0.771	AHF57914
Putative HAD superfamily hydrolase	86	13.6	3	0.786	AHF57784
Putative HAD superfamily hydrolase	1426	40.4	11	0.786	AHF57282
Arginine deiminase	4316	73.2	24	0.793	AHF57325
Putative amino acid permease	387	7.7	4	0.804	AHF57799
Serine-threonine protein phosphatase	310	28.6	5	0.807	AHF58128
Putative tRNA/rRNA methyl transferase	280	32.4	4	0.809	AHF57821
Guanosine 5'-monophosphate oxidoreductase	2694	56.6	15	0.819	AHF57936
**DNA transcription and translation/ Cell division proteins**					
Putative endonuclease	482	15.5	11	0.544	AHF58049
Putative dimethyladenosine transferase	29	9.6	3	0.553	AHF58334
Transcription regulator GntR	792	33.8	8	0.584	AHF57497
Excinuclease ABC subunit B	1459	40.4	23	0.598	AHF58183
DNA gyrase subunit B	1269	35.9	20	0.64	AHF57143

Putative deoxyribonuclease	845	48.6	12	0.662	AHF58344
Putative endoribonuclease L-PSP	1469	81.1	6	0.692	AHF57574
Putative methyltransferase	130	13.3	3	0.692	AHF57151
Uracil-DNA glycosylase	225	17.7	3	0.706	AHF58300
DNA polymerase III DnaE	286	9.3	10	0.781	AHF58105
DNA polymerase III alpha subunit	1320	22.9	26	0.771	AHF57985
Cytidylate kinase	435	44.4	7	0.787	AHF57490
Deoxyribose-phosphate aldolase	2025	69.1	11	0.782	AHF58081
Cell division protein FtsY	904	43	12	0.783	AHF57275
Formamidopyrimidine-DNA glycosylase	454	43.5	8	0.789	AHF58329
Holliday junction resolvase	288	37.6	4	0.79	AHF57660
Phosphoribosylpyrophosphate synthetase	1573	39.1	10	0.797	AHF58192
DNA ligase	1511	34	20	0.814	AHF58233
**Intracellular ionic regulation proteins**					
TrkH family potassium uptake protein,transmembrane component	103	6.2	3	0.635	AHF58226
Putative ferric uptake regulator	171	20.3	3	0.728	AHF58217
**Oxidoreduction proteins**					
C-terminal truncated oxidoreductase	179	38.9	3	0.614	AKM54520
NADH oxidase	1184	33.8	13	0.622	AHF57728
Putative oxidoreductase	563	34.3	7	0.663	AHF58103
Putative thiol peroxidase	1023	54.8	7	0.765	AHF57636
N-terminal truncated oxidoreductase	502	23.8	4	0.777	AKM53707
4-hydroxy-3-methylbut-2-en-1-yl diphosphate synthase	593	28.3	9	0.819	AHF57789
**Transferase and transport proteins**					
Lactose/cellobiose-specific PTS system IIB component	697	83.2	6	0.372	AHF57238
Lichenan-specific PTS system IIA component	270	33.6	3	0.387	AHF57244
N-acetylglucosamine-specific PTS system IICB component	526	10.3	4	0.396	AHF58319
Putative ABC-type amino acid transport system ATP-binding protein	86	20	4	0.573	AHF57787
ABC-type transport system substrate-binding protein	1643	35.3	13	0.613	AHF57211
ABC-type transport system permease protein	288	14.1	3	0.618	AHF57214
ABC-type transport system permease protein	1105	20.5	14	0.682	AHF57213
ABC-type transport system permease	523	17.1	16	0.737	AHF58317

protein					
Prolipoprotein diacylglyceryl transferase	78	5.3	3	0.746	AHF57998
Putative phosphopantetheine adenylyltransferase	297	37.9	5	0.8	AHF57395
Putative S-adenosylmethionine-dependent methyltransferase	426	28.8	7	0.803	AHF57876
Lactose phosphotransferase system repressor	660	44.4	10	0.809	AHF58120
Putative 1-acyl-sn-glycerol-3-phosphate acyltransferase	1258	35.5	9	0.829	AHF57582
**Lipoprotein and lipid metabolism proteins**					
Putative lipoprotein	31	2.8	1	0.509	AHF57559
Putative undecaprenyl pyrophosphate synthase	143	21.1	4	0.557	AHF57989
Putative lipoprotein	3307	49.9	20	0.588	AHF58304
Putative spiralin	108	9.9	3	0.664	AHF58284
Membrane-associated lipoprotein	2001	42.6	16	0.771	AHF57830
**Hypothetical proteins**					
Conserved hypothetical protein	674	41.4	6	0.173	AHF58163
Putative transmembrane protein	930	25.1	8	0.323	AHF57517
Conserved hypothetical protein	7456	43.6	29	0.337	AHF58313
Hypothetical protein	67	19.6	4	0.407	AHF57236
Hypothetical protein	233	19.4	2	0.41	AHF58241
Conserved hypothetical protein	75	5.7	2	0.486	AHF57283
Putative transmembrane protein	27	1	1	0.515	AHF57298
Putative transmembrane protein	14	1.3	1	0.516	AHF57748
Conserved hypothetical protein	441	32.2	6	0.517	AHF57624
Hypothetical protein	1170	59.7	16	0.535	AHF58014
Hypothetical protein	163	24.6	3	0.536	AHF58051
Putative transmembrane protein	141	11.9	4	0.55	AHF57948
Putative transmembrane protein	180	7.8	9	0.579	AHF57319
Putative transmembrane protein	47	2.3	1	0.588	AHF57940
Conserved hypothetical protein	761	59.1	8	0.605	AHF57640
Conserved hypothetical protein	259	7	2	0.611	AHF58299
Conserved hypothetical protein	334	37.5	10	0.62	AHF57714
Conserved hypothetical protein	1400	43.2	4	0.624	AHF57618
Conserved hypothetical protein	222	14.7	4	0.649	AHF57449
Conserved hypothetical protein	184	31.2	4	0.653	AHF58115
Conserved hypothetical protein	221	47.8	5	0.674	AHF57790
Conserved hypothetical protein	87	16.8	2	0.678	AHF58197
Conserved hypothetical protein	652	25.2	3	0.681	AHF57877
Conserved hypothetical protein	147	20.1	3	0.712	AHF57873
Conserved hypothetical protein	585	56.2	3	0.714	AHF57594

Conserved hypothetical protein	250	14.5	4	0.715	AHF57531
Conserved hypothetical protein	251	6.8	2	0.73	AHF57404
Conserved hypothetical protein	101	29.9	3	0.734	AHF57703
Hypothetical protein	129	25.4	2	0.735	AHF57472
Putative transmembrane protein	681	17.8	7	0.742	AHF57890
Hypothetical protein	112	28.6	3	0.744	AHF57209
Hypothetical protein	24	9.3	1	0.752	AHF57892
Hypothetical protein	208	18.8	4	0.755	AHF57186
Conserved hypothetical protein	304	40.1	8	0.756	AHF58172
Conserved hypothetical protein	112	5.8	4	0.757	AHF58068
Conserved hypothetical protein	646	18.1	13	0.757	AHF58288
Conserved hypothetical protein	384	44.3	11	0.788	AHF58111
Conserved hypothetical protein	106	12.8	2	0.793	AHF57824
Conserved hypothetical protein	232	11.1	1	0.793	AHF57710
Conserved hypothetical protein	922	30.8	3	0.796	AHF58171
Conserved hypothetical protein	323	22.2	2	0.795	AHF58209
Conserved hypothetical protein	470	19.7	5	0.8	AHF57591
Conserved hypothetical protein	2422	59	18	0.808	AHF57386
Conserved hypothetical protein	253	17.1	5	0.822	AHF58204
